# Chemical Composition, Antifungal and Antioxidant Activities of *Hedyosmum brasiliense* Mart. ex Miq. (Chloranthaceae) Essential Oils

**DOI:** 10.3390/medicines4030055

**Published:** 2017-07-17

**Authors:** Cynthia Murakami, Inês Cordeiro, Marcus Tullius Scotti, Paulo Roberto H. Moreno, Maria Cláudia M. Young

**Affiliations:** 1 Programa de Pós-Graduação em Biodiversidade Vegetal e Meio Ambiente, Instituto de Botânica de São Paulo, São Paulo 04301-902, Brazil; 2 Instituto de Botânica de São Paulo, São Paulo 04301-902, Brazil; isandona@uol.com.br (I.C.); marxyoungmc@gmail.com (M.C.M.Y.); 3 Laboratório de Quimioinformática, Universidade Federal da Paraíba, João Pessoa 58051-900, Brazil; mtscotti@gmail.com; 4 Instituto de Química, Universidade de São Paulo, São Paulo 05508-000, Brazil; prmoreno@iq.usp.br

**Keywords:** *Hedyosmum brasiliense*, essential oil, GC-MS, bioautography, curzerene, antioxidant, Atlantic Rain Forest

## Abstract

**Background**: *Hedyosmum brasiliense* Mart. ex Miq. (Chloranthaceae) is a dioecious shrub popularly used in Brazil to treat foot fungi and rheumatism. This work investigated the chemical composition, antifungal, and antioxidant activities of flowers and leaves of *H. brasiliense* essential oils; **Methods**: *H. brasiliense* male and female flowers and leaves were collected at Ilha do Cardoso (São Paulo) and the essential oils were extracted by hydrodistillation and analyzed by GC/MS and their similarity compared by Principal Component Analysis. Antifungal activity was performed by bioautography and antioxidant potential by 2,2-diphenyl-2-picrylhydrazyl hydrate (DPPH) free radical scavenging and β-carotene/linoleic acid system; **Results**: The major compounds for all oils were sabinene, curzerene, and carotol, but some differences in their chemical composition were discriminated by Principal Component Analysis (PCA) analysis. Bioautography showed two antifungal bands at Rf’s 0.67 and 0.12 in all samples, the first one was identified as curzerene. The oils presented stronger antioxidant potential in β-carotene/linoleic acid bioassay, with IC_50_’s from 80 to 180 μg/mL, than in DPPH assay, with IC_50_’s from 2516.18 to 3783.49 μg/mL; **Conclusions**: These results suggested that curzerene might be responsible for the antifungal activity of *H. brasiliense* essential oils. Besides, these essential oils exhibited potential to prevent lipoperoxidation, but they have a weak radical scavenger activity.

## 1. Introduction

The family Chloranthaceae includes plants grouped in four genera: *Sarcandra* Gardner, *Chloranthus* Sw., *Ascarina* J.R. Forst. and G. Forst., and *Hedyosmum* Sw. The genus *Hedyosmum* comprises 40 species of shrubs and small trees distributed from Mexico, throughout Central America to Bolivia, east of the Guianas and the Antilles [1]. The name *Hedyosmum* comes from the Greek “pleasant fragrance” and alludes to one of the most remarkable characteristics of these plants, the pleasant aromatic smell coming from all its parts [1,2]. According to Todzia [1], different *Hedyosmum* species have been popularly used all around Central and South America for different purposes: *H. scabrum* (Ruiz & Pav.) Solms for conception stimulation; *H. scabrum* and *H. scaberrimun* Standl. as stomach pain relievers; *H. sprucei* Solms for snake bites; *H. cumbalense* H. Karst. as a stimulant; and *H. racemosum* (Ruiz & Pav.) G. Don as a treatment to joint pain. The essential oil composition from some species of this genus (*H. sprucei*, *H. arborescens* Sw., *H. scabrum*, *H. colombianum* Cuatrec., *H. angustifolium* (Ruiz & Pav.) Solms, *H. mexicanum* C. Cordem., *H. bonplandianum* Mart., and *H. costaricense* C.E. Wood ex W.C. Burger) have previously been described [3,4,5,6,7,8], as well as their antioxidant and cytotoxic properties [3,4].

*Hedyosmum brasiliense* Mart. ex Miq., popularly known as “chá de bugre”, “cidreira”, or “cidrão”, is a dioecious shrub distributed throughout central and southern Brazilian regions and western Paraguay [9,10]. Its leaves have been used in folk medicine to treat migraine, ovarium dysfunction, foot fungi, rheumatism, stomach pain, and as a diuretic [1,2]. Chemical and pharmacological studies with the aerial parts of *H. brasiliense* indicated analgesic, anxiolytic, antidepressive, and aphrodisiac effects for the crude extracts and for some isolated sesquiterpene lactones [11,12,13,14,15,16]. There are also some reports on the antimicrobial activity for the ethanol extract [17] and the essential oils [18,19], however none of them could attribute the observed bioactivity to a specific compound. The essential oil presented as major compounds specimen sabinene, (Z)-β-guaiene and pinocarvone, for a São Paulo State [18], and pinocarvone, curzerene and carotol, for one from Santa Catarina State [19]. To the best of our knowledge, this is the first report on the chemical composition and biological activities of *H. brasiliense* flower essential oils. In addition, this is also the first time that the antioxidant activity is described for this species. Therefore, this work aims to investigate the chemical composition of the essential oils from *H. brasiliense* male and female flowers and leaves collected at Ilha do Cardoso (São Paulo, Brazil) and to compare their antifungal and antioxidant activities, as well as to identify the main antifungal constituents in the essential oils. 

## 2. Materials and Methods

### 2.1. Plant Material

*Hedyosmum brasiliense* Mart. ex Miq. (Chloranthaceae) flowers and leaves were collected from male and female specimens at Parque Estadual da Ilha do Cardoso, São Paulo, Brazil (25°05′ S and 47°55′ W, 14 m alt) in September of 2015. The taxonomic identity was confirmed by Dr. Inês Cordeiro (Instituto de Botânica, São Paulo, Brazil). The voucher specimens were deposited in the Herbarium at the Instituto de Botânica, São Paulo, Brazil, with the accession No. SP 475335. 

### 2.2. Extraction of the Essential Oils

Nine male and female specimens were collected. Leaves and flowers were pooled to provide representative homogeneous samples of the population, separated into three replicates and stored under refrigeration (−22 °C) until extraction. Essential oil was obtained by hydrodistillation for 3.5 h in a Clevenger-type apparatus. The crude oil was separated, dried over anhydrous sodium sulfate and stored in a glass flask at −22 °C until GC-MS analysis and biological activities. The oil yields were calculated based on the oil and fresh plant material weights as mean ± standard deviation of the triplicates [18]. 

### 2.3. GC-MS Analysis

Essential oils were solubilized in acetone 1:100 *v*/*v* (Merck KGaA, Darmstadt, Germany). Chemical analysis of the essential oils was performed on an Agilent 6890 Series GC (Agilent, Santa Clara, CA, USA), interfaced with a 5973 Series quadrupole MS detector (Agilent, Santa Clara, CA, USA) and equipped with a DB-5MS column (30 m × 0.25 mm i.d. × 0.25 μm) (Agilent J&W, Santa Clara, CA, USA). Chromatography conditions were as follows; oven temperature program: 40 °C for 1 min with subsequent temperature increase of 3 °C/min up to 240 °C, keeping it at this temperature for 10 min.; carrier gas: He at a flow rate of 1 mL/min; injector and detector temperature of 250 °C; electron ionization: 70 eV. The component abundances were expressed as the mean ± standard deviation of the triplicates. The essential oil components were identified by comparing the retention indices (evaluated in relation to the retention times of a series of *n*-alkanes) and by comparison of their mass spectra with those reported in the literature [20,21].

### 2.4. Principal Component Analysis of the Essential Oils

Principal Component Analysis (PCA) was performed using composition of *H. brasiliense* essential oils. Prior to the PCA analysis, all the variables were standardized for a normalized PCA. The Unscrambler^®^ X version 9.7 (CAMO Software, Oslo, Norway) was used to perform PCA analysis and generate score and loading plot [22].

### 2.5. Antifungal Activity

The microorganisms used in antifungal assay, *Cladosporium cladosporioides* (Fresen de Vries (CCIBt 140) and *C. sphaerospermum* Penz (CCIBt 491), have been maintained at Instituto de Botânica, São Paulo, SP, Brazil. For the antifungal assay, carried out by bioautography adapted from Homans & Fuchs method [23], 10 μL of solutions corresponding to 200, 100, 50, and 25 μg of essential oils in methanol were applied as single spots on pre-coated Thin Layer Chromatography (TLC) plates (GF_254_ silica-gel chromatoplates, Merck KGaA, Darmstadt, Germany). Nystatin, thymol, and cinnamic acid (5 µg) were used as positive controls. The plates were sprayed with a suspension of salts and glucose (6:1) containing fungus spores (> 2 × 10^6^ spores/mL) of *Cladosporium cladosporioides* and *C. sphaerospermum*. The plates were incubated in moist chambers at 27 °C for 48 h, in the dark. After the incubation period, clear inhibition zones were observed as antifungal activity of the essential oils.

The components responsible for the antifungal activities were determined by applying 400 μg of each essential oil onto three TLC plates and developed with *n*-hexane/acetone, 90:10 *v*/*v*. In one plate, the separated fraction bands were visualized with UV light (254 nm) and sprayed with Vanilin-Sulfuric Acid (VS) reagent followed by heating to 110 °C for 5 min [24]. The other two plates were used for the bioautography assay. According to the antifungal activity result, 7 mg of female flower and leaf essential oils were applied on chromatoplates, separately, and developed with the same solvent system. At the exact R_f_’s of the active bands, the plate was cut horizontally, washed with 1 mL of acetone, filtered (0.22 μm filters, Merck Millipore, Darmstadt, Germany) and further analyzed by GC-MS, at the same conditions as the essential oils [25].

### 2.6. In Vitro Antioxidant Activities

The antioxidant activity was performed by the 2,2-diphenyl-2-picrylhydrazyl hydrate (DPPH) free radical scavenging method [26], adapted for microplates. 178 μL of the essential oils solubilized in methanol at concentrations between 4480–560 μg/mL were added to a 96-well microplate and 72 μL of DPPH (0.3 mmol/L) solubilized in methanol were added. Blank reading was performed with the test sample prior to the DPPH radical incubation. As a negative control, 178 μL of methanol was used. As positive controls, quercetin from 20 to 0.625 μg/mL and *Ginkgo biloba* extract (Herbarium Laboratório Botânico S.A., Paraná, Brazil) from 40 to 1.25 μg/mL were used. The microplate was incubated in the dark for 30 min at room temperature. Then, the absorbance was measured at a wavelength of 518 nm using a multi-well scanning spectrophotometer (BIOTEK KC4, Winooski, VT, USA). The absorbance was converted to percentage of antioxidant activity (AA) using the formula AA% = 100 − {[(Abs _sample_ − Abs _blank_) × 100]/Abs _control_}. All samples were tested in triplicate and the results are expressed by mean ± standard deviation. The amount of essential oil required to reduce the initial DPPH concentration in the reaction by 50% is referred to as the inhibitory concentration (IC_50_). 

The antioxidant activity was also evaluated by the β-carotene bleaching test, which is based on spectrophotometric measurements of the β-carotene oxidation induced by the lipoperoxidation products from linoleic acid [27] to determine the concentration providing 50% inhibition (IC_50_). The essential oil (10 μL) solubilized in methanol at concentrations between 10,400–325 μg/mL was added in a 96-well microplate, together with 250 μL of a reactive solution composed by β-carotene and linoleic acid in water saturated with O_2_. Methanol (10 μL) was used as the negative control and as the positive controls, butylhydroxytoluene (BHT) and butylhydroxyanisole (BHA) at concentrations between 130–4.1 μg/mL. The microplate was incubated in the dark at 45 °C and subsequently the absorbance was read at 450 nm (multi-well scanner BIOTEK KC4, Winooski, VT, USA), immediately after adding the reactive solution and every 30 min for 2 h. The absorbance was converted to percentage of antioxidant activity (AA) using the AA% = 100 − {[(Abs _final_ − Abs _initial_) × 100]/Abs _control_}. All samples were tested in triplicate and the results are expressed by mean ± standard deviation.

## 3. Results

### 3.1. Chemical Composition of the Essential Oil

The essential oil yields for male and female *H. brasiliense* flowers were 0.24 ± 0.01 and 0.38 ± 0.01%, respectively. The yield of leaf essential oils, both male and female, presented a yield of 0.33 ± 0.01% (Table 1). 

Fifty compounds were identified from all essential oils analyzed, comprising 96–98% of the total components. The major compounds for all *H. brasiliense* essential oils were sabinene, curzerene, and carotol. Leaf essential oils presented mostly monoterpenes (53% ♂ and 55% ♀), followed by sesquiterpenes (39% ♂ and 41% ♀), and phenylpropanoids (2% ♀ and 5% ♂). Flower essential oils presented monoterpenes (54% ♀ and 45% ♂), sesquiterpenes (40% ♂ and 50% ♀), and phenylpropanoids (1% ♂ and 2% ♀). The leaf main constituents were sabinene (16%), β-pinene (5%), 1,8-cineole (3–7%), methyl eugenol (2–5%), curzerene (17–18%), and carotol (6%), while flowers presented sabinene (8–10%), α-phellandrene (1–8%), 1,8-cineole (2–7%), germacrene D (4–6%), curzerene (11%), and carotol (9%) as major compounds. 

**Figure 1:**
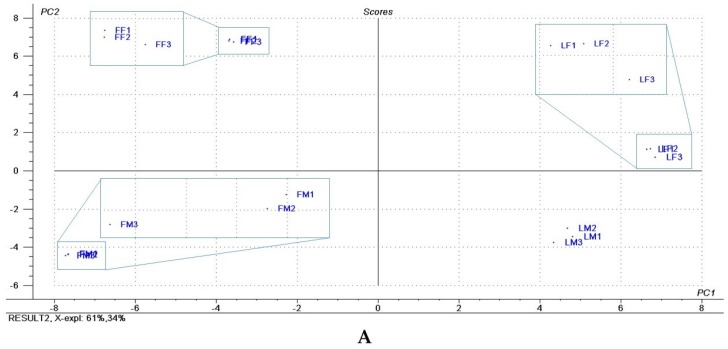

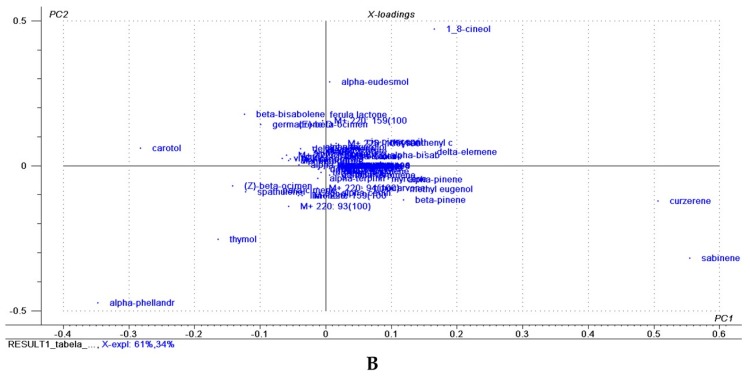
(**A**) Scores and (**B**) Loadings of Principal Component Analysis (PCA) of the chemical composition of *Hedyosmum brasiliense* essential oils; FF 1, 2, 3 = female flowers; FM 1, 2, 3 = male flowers; LF l, 2, 3 = female leaves; LM l, 2, 3 = male leaves.

Principal Component Analysis (PCA) data from Table 1 generated a plot, which condensed scores and loadings of the first two components, PC1 and PC2, which explain 61% and 34% of total variance (Figure 1). Leaves presented higher amounts of sabinene and curzerene than flowers, while flowers presented higher amounts of carotol, as discriminated by PCA (Figure 1). Furthermore, PCA analysis showed that female flowers and leaves presented higher amounts of 1,8-cineole than the male counterparts (Figure 1). 

### 3.2. Antifungal Activity

**Figure 2:**
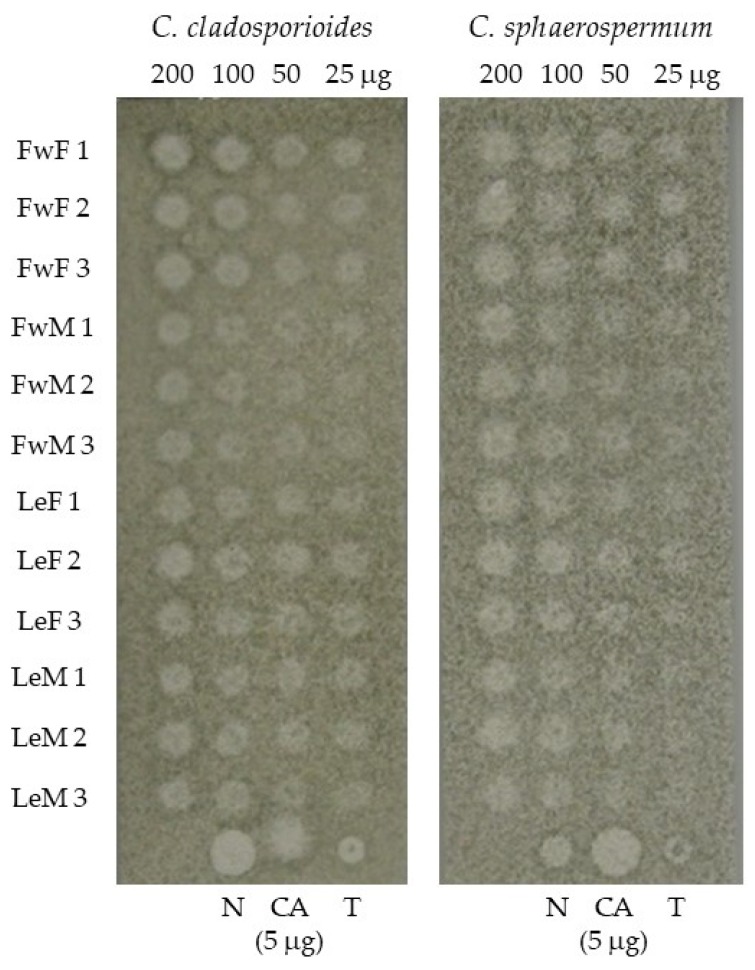
Preliminary antifungal activity of *Hedyosmum brasiliense* essential oils against *Cladosporium cladosporioides* and *C. sphaerospermum*. FwF = female flowers; FwM = male flowers; LeF = female leaves; LeM = male leaves; N = nystatin; CA = cinnamic acid; T = thymol.

The essential oils of *H. brasiliense* presented clear inhibition zones corresponding to antifungal activity in the limit of detection against *Cladosporium cladosporioides* and *C. sphaerospermum* (Figure 2). Female flowers presented the strongest activity for both fungi from 200 μg until 25 μg of essential oil tested, but the other oils presented weaker inhibition, up to 50 μg against *C. sphaerospermum* and to 25 μg against *C. cladosporioides*. 

**Figure 3:**
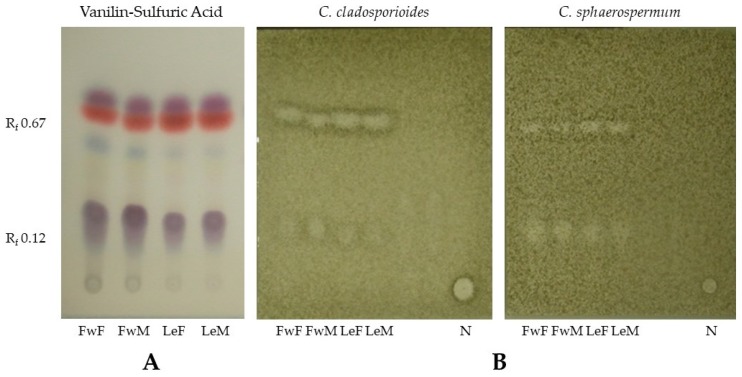
(**A**) Detection of terpenes of *H. brasiliense* essential oils by Vanilin-Sulfuric Acid (VS) spray reagent followed by heating to 110 °C; (**B**) Antifungal activity of *Hedyosmum brasiliense* essential oils, developed with *n*-hexane/acetone (90:10, *v*/*v*), against *Cladosporium cladosporioides* and *C. sphaerospermum*. R_f_ 0.67 = strong activity; R_f_ 0.12 = weak activity; FwF = female flowers; FwM = male flowers; LeF = female leaves; LeM = male leaves; N = nystatin.

**Figure 4:**
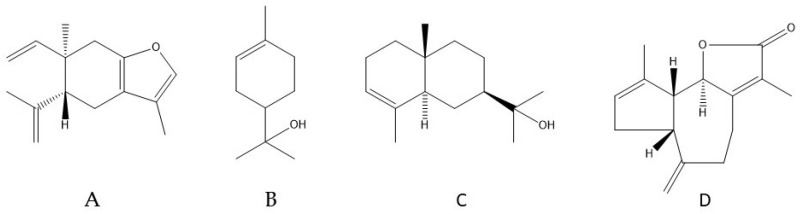
Chemical structures of the main compounds found in the active fractions of *H. brasiliense* essential oils: curzerene (**A**); α-terpineol (**B**); α-eudesmol (**C**); and ferula lactone I (**D**).

After TLC elution of the most active oils, two inhibition bands, at retention factors (R_f_’s) of 0.67 and 0.12, were observed in all samples (Figure 3). After scraping the active bands, the GC-MS analysis indicated that curzerene (Table 2, Figure 4) was the main compound for the R_f_ 0.67 fractions, comprising 97% and 82% for both flower and leaf oils. The band at R_f_ 0.12 was composed by a complex mixture, with predominance of α-terpineol, α-eudesmol, and ferula lactone I (Figure 4) in different proportions, not allowing to point only one single active compound, as presented in Table 2.

### 3.3. Antioxidant Activity

Antioxidant activity was determined by 2,2-diphenyl-1-picrylhydrazyl (DPPH) free radical scavenging and β-carotene bleaching methods. The 50% inhibitory concentration (IC_50_) were calculated and the IC_50_ values are presented in Table 3. Quercetin and *Ginkgo biloba* extract were employed as positive controls showing IC_50_ 2.5 ± 0.09 and 13.5 ± 0.5 μg/mL, respectively, for DPPH assay, while butylhydroxytoluene (BHT) and butylhydroxyanisole (BHA) were the positive controls for β-carotene assay, showing IC_50_ 0.08 ± 0.09 and 0.27 ± 0.02 μg/mL, respectively.

## 4. Discussion

Although there were previous studies on the essential oils from *H. brasiliense* leaves, none of them discriminated among male and female plants [18,19,28] and, to the best of our knowledge, this is the first report with flower essential oils. In our study, the essential oil yields were similar for flowers and leaves of *H. brasiliense*, with the male flowers presenting the lowest oil amounts. Regarding the leaf oil contents, the results were like those found for a Paraná State specimen (Brazil) [28], collected in an area not far from Ilha do Cardoso (São Paulo State, Brazil). However, these values were lower than those found for a specimen collected in Santa Catarina State (Brazil), whose leaves yielded 0.5% (*v*/*w*) [19], but this place is further south from our collection site.

The essential oil analysis for the Parana (Brazil) specimen was not complete, most of the oil components were not identified [28]. Nonetheless, with the composition published it was possible to detect 23 common compounds with the São Paulo (Brazil) specimen, but only sabinene (7%) was a common major compound. However, for the Santa Catarina (Brazil) study were found 22 compounds in common [19], having as major compounds α-terpineol (10%), curzerene (9%), pinocarvone (8%), β-thujene (7%), and carotol (6%). Curzerene (Figure 4A) was the only common major compound with the present analysis, but with 17–18% in the leaves and 11% in flowers. 

The essential oils from other *Hedyosmum* species leaves (*H. mexicanum*, *H. bonplandianum*, *H. arborescens,* and *H. angustifolium*) have also presented high amounts of sabinene in their composition [4,7,8], as well as β-pinene, pinocarvone, and curzerene in the essential oil from *H. colombianum* leaves [6], similarly to our study.

*Hedyosmum* spp essential oils are known to contain several phenylpropanoids [3,5,6], sometimes in high amounts, as in essential oil from *H. scabrum* leaves, which contained 55.8% of estragole and 6.6% of anethole [5]. Also, some *H. brasiliense* specimens from different geographic origins have shown methyl chavicol, eugenol acetate, methyl eugenol, and estragole [18,19,28]. However, the only phenylpropanoid detected in our study was methyl eugenol, but in small amounts. 

PCA plot shows that leaves have a higher percentage of sabinene and curzerene than flowers, which have a more significant amount of carotol, responsible for differentiating them. Besides that, PCA was also able to discriminate male flowers and leaves from the female ones by the 1,8-cineole abundance, which was higher in the female counterparts (Table 1 and Figure 1). These results showed that it is important to analyze separately male and female individuals, part of the variable results on the chemical composition might be explained by not separating the plant genders. 

*Cladosporium* spp. are phytopathogenic filamentous fungi usually chosen for bioautographic assays, as they present high sensitivity and permit the detection of fungitoxic substances by contrast with their dark color [23]. Preliminary assays for the detection limit with bioautography confirmed the antifungal activity for the *H. brasiliense* specimen from São Paulo against *C. cladosporioides* and *C. sphaerospermum*, with the highest activity for the female flower essential oil (Figure 2). In a previous study, the antimicrobial activity of *H. brasiliense* leaf essential oils has already been confirmed for Gram-positive bacteria and fungi such as the dermatophytes *Microsporum canis*, *M. gypseum*, *Trichophyton mentagrophytes,* and *T. rubrum* and the yeasts *Candida albicans* and *C. parapsilosis* [19].

The bioautography followed by TLC separation has been useful to determine antimicrobial activity of essential oils and to isolate the active compounds. A bioautography study with five aromatic plants (*Thymus vulgaris*, *Lavandula angustifolia* Chaix, *Eucalyptus globulus* Labill., *Mentha spicata* L., and *Cinnamomum zeylanicum* Blume) against five bacteria (*Pseudomonas syringae*, *Xanthomonas campestris*, *Staphylococcus epidermidis*, *S. saprophyticus*, and *S. aureus*) allowed to attribute the activity of *Thymus vulgaris* essential oil to thymol, comparing to a pure standard, as well as the activity of *C. zeylanicum* essential oil to eugenol, by the same principle [29]. A bioautography assay with *Mentha* x *piperita* L. essential oil against *Candida albicans*, followed by TLC separation of the active fraction, indicated that menthol was responsible for this activity [25]. Also, Guerrini et al. [3] detected α-cadinol, α-muurolol, τ-muurolol, and linalool in the active fraction with antibacterial activity against *S. aureus* using High Performance Thin Layer Chromatography (HPTLC) plates for separation of *H. sprucei* essential oil. In our study, the bioautography methodology indicated curzerene as the main compound for the active band found at R_f_’s 0.67 and α-terpineol, α-eudesmol and ferula lactone I for the active band at R_f_ 0.12 (Table 2). The *H. brasiliense* leaf essential oil collected at Santa Catarina (Brazil) presented antifungal activity and it was also rich in curzerene (8.9%) and α-terpineol (10.2%) (Figure 4 A,B) [25], supporting our results. 

Curzerene has been known for its toxic effects against *Anopheles subpictus* (LC_50_ = 4.14 μg/mL), *Aedes albopictus* (LC_50_ = 4.57 μg/mL), and *Culex tritaeniorhynchus* (LC_50_ = 5.01 μg/mL) [30]. In addition, curzerene also showed antiproliferative effects in SPC-A1 human lung carcinoma, with IC_50_ = 47 μmol/L in 72 h (in vitro) and 135 mg/kg daily (in vivo) [31]. *Eugenia uniflora* L. essential oil, rich in curzerene (42.6%), showed antifungal activity, inhibiting completely *Paracoccidioides Braziliensis* at 62.5 μg/mL [32]. Moreover, another *E. uniflora* essential oil composed by 47.3% of curzerene presented anti-*Leishmania* activity, with IC_50_ = 3.04 μg/mL against promastigotes and IC_50_ = 1.92 μg/mL against amastigotes of *L. amazonensis* [33]. 

The antioxidant activity of essential oils is already well known [34,35,36,37]. According to Karadag et al. [38], the antioxidant potential is related to compounds capable of protecting a biological system against the potentially harmful effect of reactive oxygen species. The results of a single-assay can give only a narrow view of the essential oil antioxidant properties. Therefore, the antioxidant potential of *H. brasiliense* essential oils was determined by two different mechanisms of action: DPPH free radical scavenging, that works as electron transfer, and β-carotene/linoleic acid assay, measuring the suppression of lipoperoxides. In the DPPH assay, the IC_50_ values for all the oils ranged from 2516.18 to 3783.49 μg/mL (Table 2), indicating a weak electron transfer capacity for the oils when compared to the positive controls quercetin and *G. biloba* extract, that presented IC_50_ values of 2.5 and 13.5 μg/mL, respectively. These results are comparable to those obtained for *Salvia tomentosa* Mill. essential oils [36], which was rich in β-pinene (39.7%), α-pinene (10.9%) and camphor (9.7%), and it was not considered effective as antioxidant by DPPH and β-carotene/linoleic acid assay. Similarly, the essential oils of seven *Artemisia* spp. were tested by the same methods and showed weak activities, as their composition were markedly rich in non-phenolic components [39]. The antioxidant capacity of essential oils, by the DPPH method normally, is not very high when compared with that obtained for extracts and fractions rich in phenolic compounds. The major antioxidative plant phenolic compounds known are eugenol, carvacrol, thymol, menthol, and safrole, among others [34]. In a test with 98 pure essential oil components for their antioxidant activity, phenols, such as thymol and carvacrol, were the most active compounds, followed by alcohols mono- and sesquiterpenes and ketones α,β-insaturated, however hydrocarbons presented very low antioxidant activity [40]. On the other hand, our results indicated that the essential oils of *H. brasiliense* were much more active in the β-carotene bleaching assay, presenting IC_50_ values from 71.12 to 180.71 μg/mL (Table 2), indicating their ability in destroying the conjugated diene hydroperoxides, the end products of linoleic acid peroxidation. In this way, the low radical scavenging activity observed for the *H. brasiliense* essential oils might be explained by the low amounts of phenolic compounds present in the oil, only 1.11 to 3.63% of thymol, and the high amounts of mono- and sesquiterpene hydrocarbons (Table 1). Since antioxidant activity of the entire oil is the result of the interaction of all constituents [41], it is hard to attribute the essential oils activity to a single compound, as other oil components may contribute exhibiting synergistic or antagonistic effects [42]. 

## 5. Conclusions

The leaf and flower essential oils were dominated by sabinene, 1,8-cineole, curzerene, and carotol. However, the contents of these constituents differed according to the plant part and gender.

This was the first report of antifungal activity of curzerene, explaining partially the activity of *H. brasiliense* essential oils by bioautography. Moreover, the complex mixture of α-terpineol, α-eudesmol, and ferula lactone I may have acted synergistically to the antifungal activity observed. Bioautography assay helped to detect antifungal fractions in a complex matrix of essential oils and guided to the isolation of target-directed constituents. Further studies are necessary to isolate higher amounts of pure curzerene, in order to test its limit of detection. 

Antioxidant activity of essential oils from *H. brasiliense* was more effective by β-carotene discoloration assay than by DPPH assay, indicating their higher ability in quenching conjugated hydroperoxides than free radicals. The inhibition of lipid peroxidation may have been a result of the interaction of all constituents of the essential oils, since it was not possible to attribute antioxidant activity to a single compound. 

## Figures and Tables

**Table 1:** Chemical composition of *Hedyosmum brasiliense* essential oils.

Compound	RI	RI_ref_ (a)	Relative Amount (%)			
			♂ Flowers	♀ Flowers	♂ Leaves	♀ Leaves
4-hydroxy-4-methyl-2-pentanone	841	839	1.58 ± 0.02	1.44 ± 0.03	1.53 ± 0.06	1.49 ± 0.02
α-pinene	933	939	2.23 ± 0.02	1.96 ± 0.02	3.16 ± 0.10	3.66 ± 0.11
sabinene	972	975	9.46 ± 0.04	8.05 ± 0.11	15.81 ± 0.19	15.81 ± 0.17
β-pinene	976	979	3.91 ± 0.03	2.73 ± 0.04	4.64 ± 0.13	5.21 ± 0.19
myrcene	990	990	1.75 ± 0.02	1.45 ± 0.02	2.48 ± 0.05	2.79 ± 0.14
α-phellandrene	1003	1002	8.14 ± 0.07	1.10 ± 0.01	2.79 ± 0.44	0.82 ± 0.06
α-terpinene	1016	1017	1.07 ± 0.01	0.91 ± 0.01	1.18 ± 0.12	1.09 ± 0.12
ρ-cymene	1023	1024	1.51 ± 0.01			
limonene	1028	1029	2.53 ± 0.01	1.08 ± 0.01	1.68 ± 0.06	1.46 ± 0.05
1,8-cineole	1031	1031	1.65 ± 0.01	7.22 ± 0.07	3.44 ± 0.04	6.86 ± 0.17
(Z)-β-ocymene	1036	1037	2.26 ± 0.01	0.66 ± 0.01		
*(E)*-β-ocymene	1048	1050	1.85 ± 0.01	3.00 ± 0.04	0.90 ± 0.06	1.91 ± 0.01
γ-terpinene	1059	1059	1.76 ± 0.01	1.44 ± 0.01	1.81 ± 0.16	1.68 ± 0.17
terpinolene	1082	1088			0.57 ± 0.00	
linalool	1097	1096	1.53 ± 0.01	1.5 ± 0.01	0.99 ± 0.03	1.31 ± 0.03
camphor	1146	1146		0.73 ± 0.01		
pinocarvone	1161	1164	3.65 ± 0.02	3.15 ± 0.02	4.49 ± 0.11	3.94 ± 0.07
terpinen-4-ol	1177	1177	3.34 ± 0.02	3.11 ± 0.04	3.30 ± 0.36	3.09 ± 0.32
α-terpineol	1191	1188	1.07 ± 0.01	1.37 ± 0.01	0.61 ± 0.07	0.76 ± 0.06
citronellol	1224	1225			0.60 ± 0.03	
chrysanthenyl cis-acetate	1255	1265		1.21 ± 0.01	1.03 ± 0.08	1.55 ± 0.03
bornyl acetate	1279	1288		0.58 ± 0.00		
thymol	1285	1290	3.63 ±0.05		1.11 ± 0.20	
pinocarvyl cis-acetate	1303	1312		1.16 ± 0.01	0.72 ± 0.03	1.05 ± 0.02
δ-elemene	1327	1338		1.33 ± 0.01	2.24 ±0.11	2.30 ± 0.07
α-terpinyl acetate	1344	1349	1.47 ± 0.01	0.75 ± 0.01	0.93 ± 0.04	1.19 ± 0.02
α-copaene	1370	1376	0.71 ± 0.00	0.66 ± 0.00		
β-elemene	1384	1390	1.27 ± 0.01	1.68 ± 0.01	1.43 ± 0.09	1.58 ± 0.05
methyl eugenol	1394	1403	1.41 ± 0.02	1.96 ± 0.02	4.53 ± 0.21	1.85 ± 0.17
*(E)*-caryophyllene	1412	1419	0.65 ± 0.01	0.69 ± 0.00		
γ-elemene	1423	1436	0.69 ± 0.00	0.72 ± 0.01	0.70 ± 0.06	0.76 ± 0.02
α-humulene	1449	1454	0.80 ± 0.02	0.72 ± 1.36	0.59 ± 0.04	0.58 ± 0.00
germacrene D	1477	1485	4.23 ± 0.04	5.76 ± 0.02	3.74 ± 0.21	3.35 ± 0.13
curzerene	1489	1499	11.09 ± 0.11	11.68 ± 0.05	17.02 ± 0.66	17.78 ± 0.30
β-bisabolene	1502	1505	0.97 ± 0.01	2.66 ± 0.01		
δ-cadinene	1511	1523	0.98 ± 0.01	1.51 ± 0.01	0.63 ± 0.02	0.71 ± 0.02
(Z)-α-bisabolene	1539	1507	0.63 ± 0.01	0.95 ± 1.36	0.80 ± 0.11	1.86 ± 0.04
elemol	1542	1549		0.61 ± 0.00		
N.I. 1	1556		1.17 ± 0.01	1.66 ± 0.01	0.86 ± 0.01	0.80 ± 0.02
spathulenol	1570	1578	4.53 ± 0.04	3.08 ± 0.01	3.05 ± 0.44	2.23 ± 0.08
viridiflorol	1578	1592	0.81 ± 0.01	0.82 ± 0.00		
N.I. 2	1587		0.67 ± 0.00	0.86 ± 0.01		
carotol	1596	1594	9.72 ± 0.04	9.36 ± 0.05	6.48 ± 0.10	5.91 ± 0.15
N.I. 3	1620			0.78 ±0.01		0.75 ± 0.03
epi-α-cadinol	1626	1640	0.93 ± 0.01		0.96 ± 0.17	
daucol	1636	1642	0.61 ± 0.00			
N.I. 4	1641		0.74 ± 0.01		0.84 ± 0.09	
α-muurolol	1643	1646		0.74 ± 0.08		
α-eudesmol	1648	1653	1.66 ± 0.02	3.08 ± 0.02	1.06 ± 0.04	1.79 ± 0.08
(Z)-α-santalol	1661	1675		0.57 ± 0.00		
ferula lactone I	2001	1974	1.75 ± 0.02	3.70 ± 0.02	1.88 ± 0.69	2.48 ± 0.70
Monoterpene hydrocarbon (%)			38.12 ± 0.09	29.6 ± 0.27	38.08 ± 1.44	41.28 ± 0.97
Oxygenated monoterpene (%)			16.27 ± 0.06	15.00 ± 0.10	15.33 ± 0.60	14.38 ± 0.46
Sesquiterpene hydrocarbon (%)			10.93 ± 0.06	16.68 ± 0.05	9.93 ± 0.74	10.75 ± 0.50
Oxygenated sesquiterpene (%)			29.03 ± 0.19	33.45 ± 0.31	29.39 ± 1.61	30.18 ± 1.21
Phenylpropanoid (%)			1.41 ± 0.02	1.96 ± 0.02	4.53 ± 0.21	1.85 ± 0.17
Not identified (%)			4.24 ± 0.04	3.30 ± 0.01	2.76 ± 0.13	1.56 ± 0.02
Total identified (%)			95.75 ± 0.04	96.69 ± 0.01	97.26 ± 0.11	98.44 ± 0.02
Yield (%)			0.24 ± 0.01	0.38 ± 0.01	0.33 ± 0.01	0.33 ± 0.01

RI = Retention indices on DB-5 column; RI_ref_ (a) = [20]; ♂ = male; ♀ = female; NI 1: M+ 220: 159(100) 119(99) 145(66) 105(53) 131(42); NI 2: M+ 222: 109(100) 161(91) 107(90) 43(88) 105(86); NI 3: M+ 220: 119(100) 91(84) 105(82) 93(75) 162(59); NI 4: M+ 220: 208(100) 161(93) 193(86) 119(51) 105(49).

**Table 2:** Chemical composition of the active antifungal fractions of *H. brasiliense* female flowers and leaves essential oils isolated by bioautography-guided TLC.

Compound	RI	RI_ref_ (a)	FwF (%)		LeF (%)	
			R_f_ 0.67	R_f_ 0.12	R_f_ 0.67	R_f_ 0.12
1,8-cineole	1031	1031			12.69	
terpinolene	1082	1088		2.24		4.65
dehydro-sabina ketone	1121	1120		1.25		2.72
cis-β-terpineol	1143	1144		0.89		
2-(1Z)-propenyl-phenol	1146	1150		0.73		
cis-chrysanthenol	1162	1164		0.89		
terpinen-4-ol	1177	1177		1.87		
α-terpineol	1191	1188		9.77		10.45
δ-elemene	1327	1338			0.99	
β-elemene	1384	1390			0.92	
germacrene D	1477	1485	2.98		1.45	
curzerene	1489	1499	97.02		82.08	
δ-amorphene	1508	1512		1.50		
elemol	1542	1549		3.80		3.81
spathulenol	1570	1578		9.42		4.99
viridiflorol	1578	1592		4.35		6.57
epi-α-cadinol	1636	1640		0.80		
epi-α-muurolol	1640	1642		4.75		5.12
α-eudesmol	1648	1653		20.95		22.53
valerianol	1651	1658		2.23		
atractylone	1652	1658			1.87	
germacra-4(15),5,10(14)-trien-1-α-ol	1675	1686		0.71		
ferula lactone I	2001	1974		21.83		28.29
**Intensity of antifungal activity**			**strong**	**weak**	**strong**	**weak**
**Total identified (%)**			**100**	**87.98**	**100**	**89.13**

RI = Retention indices on DB-5 column; ref. RI_ref_ (a) = [20]; fractions R_f_ 0.67 = strong antifungal activity; fractions R_f_ 0.12 = weak antifungal activity.

**Table 3:** Antioxidant activities (IC_50_, mean ± SD) by DPPH free radical scavenging and β-carotene/linoleic acid methods of the essential oils from flowers and leaves of *H. brasiliense*.

Samples	IC_50_ (μg/mL)	
	DPPH	β-carotene/linoleic acid
♀ Flowers	3162.79 ± 40.72	180.71 ± 22.95
♂ Flowers	2516.18 ± 131.87	113.46 ± 11.38
♀ Leaves	3542.01 ± 45.31	71.12 ± 9.07
♂ Leaves	3783.49 ± 132.76	86.47 ± 18.63
Quercetin	2.50 ± 0.09	-
*Ginkgo biloba*	13.50 ± 0.50	-
BHT	-	0.08 ± 0.09
BHA	-	0.27 ± 0.02

DPPH = 2,2-diphenyl-1-picrylhydrazyl; BHT = butylhydroxytoluene; BHA = butylhydroxyanisole; ♂ = male; ♀ = female.

## Supplementary Materials

The following are available online at www.mdpi.com/2305-6320/4/3/55/s1, Figure S1: Mass spectrum of Non-identified compound 1 (N.I. 1) detected in the essential oil of *H. brasiliense* from Ilha do Cardoso; Figure S2: Mass spectrum of Non-identified compound 2 (N.I. 2) detected in the essential oil of *H. brasiliense* from Ilha do Cardoso; Figure S3: Mass spectrum of Non-identified compound 3 (N.I. 3) detected in the essential oil of *H. brasiliense* from Ilha do Cardoso; Figure S4: Mass spectrum of Non-identified compound 4 (N.I. 4) detected in the essential oil of *H. brasiliense* from Ilha do Cardoso.
