# Supplementary material for: Chemical Composition, Antifungal and Antioxidant Activities of Hedyosmum brasiliense Mart. ex Miq. (Chloranthaceae) Essential Oils
Source: Medicines (Basel). 2017 Jul 17;4(3):55. doi: 10.3390/medicines4030055 (PMC5622390; doi:10.3390/medicines4030055)
Supplement: Supplementary file 1 [file medicines-04-00055-s001.pdf]

Supplementary Materials: Chemical Composition, Antifungal and Antioxidant Activities of *Hedyosmum brasiliense* Mart. ex Miq. (Chloranthaceae) Essential Oils

Cynthia Murakami, Inês Cordeiro, Marcus Tullius Scotti, Paulo Roberto H. Moreno and Maria Cláudia M. Young

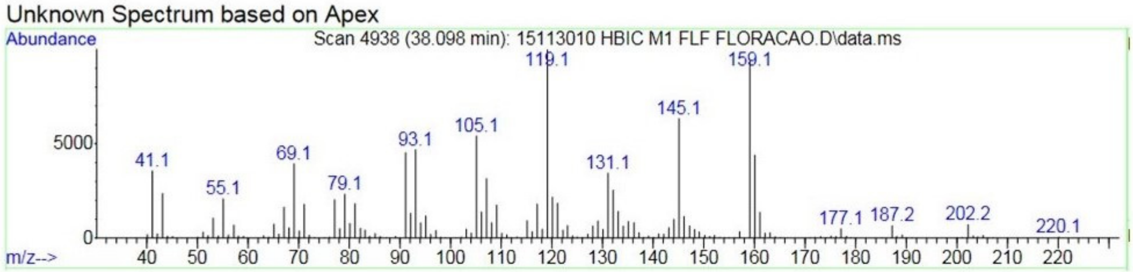

Figure S1. Mass spectrum of Non-identified compound 1 (N.I. 1) detected in the essential oil of *H. brasiliense* from Ilha do Cardoso.

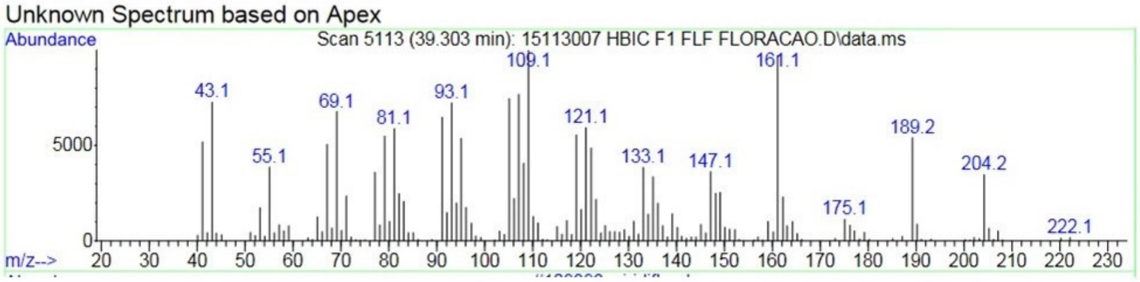

Figure S2. Mass spectrum of Non-identified compound 2 (N.I. 2) detected in the essential oil of *H. brasiliense* from Ilha do Cardoso.

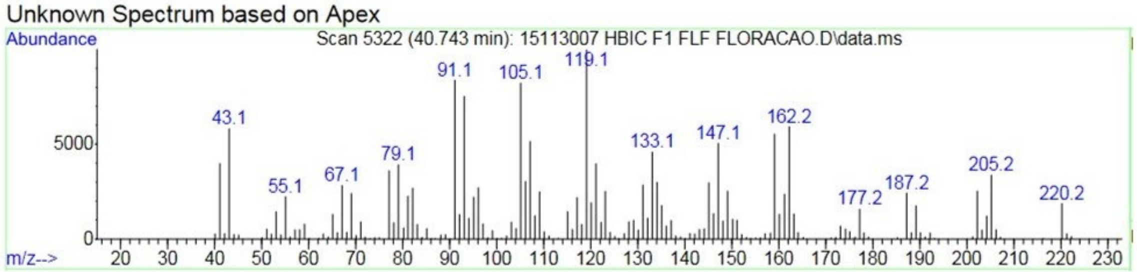

Figure S3. Mass spectrum of Non-identified compound 3 (N.I. 3) detected in the essential oil of *H. brasiliense* from Ilha do Cardoso.

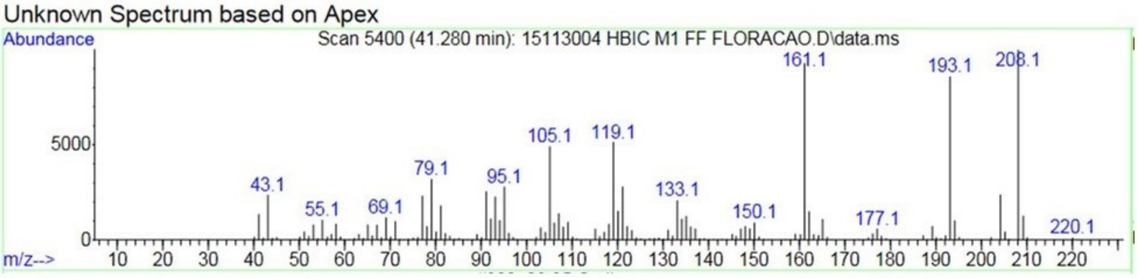

Figure S4. Mass spectrum of Non-identified compound 4 (N.I. 4) detected in the essential oil of *H. brasiliense* from Ilha do Cardoso.
